# Complete Multilineage CD4 Expression Defect Associated With Warts Due to an Inherited Homozygous CD4 Gene Mutation

**DOI:** 10.3389/fimmu.2019.02502

**Published:** 2019-11-08

**Authors:** Rosa Anita Fernandes, Martin Perez-Andres, Elena Blanco, Maria Jara-Acevedo, Ignacio Criado, Julia Almeida, Vitor Botafogo, Ines Coutinho, Artur Paiva, Jacques J. M. van Dongen, Alberto Orfao, Emilia Faria

**Affiliations:** ^1^Allergy and Clinical Immunology Department, Centro Hospitalar e Universitário de Coimbra, Coimbra, Portugal; ^2^Department of Medicine, Cancer Research Centre (IBMCC, USAL-CSIC), Cytometry Service (NUCLEUS), University of Salamanca (USAL), Salamanca, Spain; ^3^Institute of Biomedical Research of Salamanca (IBSAL), Salamanca, Spain; ^4^Biomedical Research Networking Centre on Cancer-CIBER-CIBERONC (CB16/12/00400), Institute of Health Carlos III, Madrid, Spain; ^5^Sequencing DNA Service, NUCLEUS, University of Salamanca, Salamanca, Spain; ^6^Dermatology Department, Centro Hospitalar e Universitário de Coimbra, Coimbra, Portugal; ^7^Flow Cytometry Unit—Clinical Pathology Department, Centro Hospitalar e Universitário de Coimbra, Coimbra, Portugal; ^8^Ciências Biomédicas Laboratoriais, ESTESC-Coimbra Health School, Instituto Politécnico de Coimbra, Coimbra, Portugal; ^9^Faculty of Medicine, Coimbra Institute for Clinical and Biomedical Research (iCBR), University of Coimbra, Coimbra, Portugal; ^10^Department of Immunohematology and Blood Transfusion, Leiden University Medical Center, Leiden, Netherlands

**Keywords:** CD4, warts, double-negative T-cells (DNTs), CD4 lymphopenia, idiopathic CD4 lymphocytopenia

## Abstract

Idiopathic T-CD4 lymphocytopenia (ICL) is a rare and heterogeneous syndrome characterized by opportunistic infections due to reduced CD4 T-lymphocytes (<300 cells/μl or <20% T-cells) in the absence of HIV infection and other primary causes of lymphopenia. Molecular testing of ICL has revealed defects in genes not specific to CD4 T-cells, with pleiotropic effects on other cell types. Here we report for the first time an absolute CD4 lymphocytopenia (<0.01 CD4^+^ T-cells/μl) due to an autosomal recessive CD4 gene mutation that completely abrogates CD4 protein expression on the surface membrane of T-cells, monocytes, and dendritic cells. A 45-year-old female born to consanguineous parents consulted because of exuberant, relapsing, and treatment-refractory warts on her hands and feet since the age of 10 years, in the absence of other recurrent infections or symptoms. Serological studies were negative for severe infections, including HIV 1/2, HTLV-1, and syphilis, but positive for CMV and EBV. Blood analysis showed the absence of CD4^+^ T-cells (<0.01%) with repeatedly increased counts of B-cells, naïve CD8^+^ T-lymphocytes, and particularly, CD4/CD8 double-negative (DN) TCRαβ^+^ TCRγδ^−^ T-cells (30% of T-cells; 400 cells/μl). Flow cytometric staining of CD4 using monoclonal antibodies directed against five different epitopes, located in two different domains of the protein, confirmed no cell surface membrane or intracytoplasmic expression of CD4 on T-cells, monocytes, and dendritic cells but normal soluble CD4 plasma levels. DN T-cells showed a phenotypic and functional profile similar to normal CD4^+^ T-cells as regards expression of maturation markers, T-helper and T-regulatory chemokine receptors, TCRvβ repertoire, and *in vitro* cytokine production against polyclonal and antigen-specific stimuli. Sequencing of the *CD4* gene revealed a homozygous (splicing) mutation affecting the last bp on intron 7–8, leading to deletion of the juxtamembrane and intracellular domains of the protein and complete abrogation of CD4 expression on the cell membrane. These findings support previous studies in CD4 KO mice suggesting that surrogate DN helper and regulatory T-cells capable of supporting antigen-specific immune responses are produced in the absence of CD4 signaling and point out the need for better understanding the role of CD4 on thymic selection and the immune response.

## Background

CD4 is a monomeric type I transmembrane glycoprotein consisting of four immunoglobulin-like extracellular domains connected by a short stalk to a transmembrane domain and a short cytoplasmic tail (1–4). Although rare polymorphisms have been described in humans that suppress reactivity with the anti-CD4 OKT4 antibody clone (5, 6), comparison of CD4 sequences from different animal species indicates that the basic structure of this molecule was highly preserved during evolution for more than 400 million years (7). The CD4 molecule is mostly known because it has long been used to define helper T-cells and, more recently, regulatory T-cells (Tregs). These represent two functionally unique T-cell populations responsible for driving humoral and cytotoxic responses through production of different cytokine profiles (8, 9) and suppressing the immune response (10), respectively. In addition, lower CD4 expression is also detected on antigen-presenting cells such as monocytes and dendritic cells (DCs) (11) and on megakaryocytic precursors (12).

Multiple studies have demonstrated that CD4 serves as a co-receptor during T-cell receptor (TCR) recognition of major histocompatibility complex MHC/HLA class II–associated peptides (1–3). Binding of the membrane-distal D1 domain of CD4 to non-polymorphic residues of MHC/HLA class II molecules provides a more potent stimulus for the T-cell than simply ligating the TCR alone (1–3). Thus, ligation of CD4 to MHC/HLA class II has been shown to induce positive selection of helper T-cells during thymic differentiation (4) and supports activation of helper T-cells and Tregs in blood and other lymphoid and non-lymphoid tissues (1–3). However, CD4–MHC/HLA class II affinity is low, leading to weak binding between the two proteins (2, 3). As a consequence, other molecules are required for productive interactions with downstream effects. In fact, it has been confirmed in murine models that differentiation of helper T-cells can occur in the absence of CD4 expression, suggesting that signaling via this co-receptor might be dispensable (13, 14). Thus, in CD4 knock-out (KO) mice, an expanded subpopulation of CD4/CD8 double-negative (DN) TCRαβ^+^ T-cells with T-helper ability is generated at abnormally high numbers, which functionally replace conventional CD4^+^ T-cells (13, 14).

A limited number of cases (*n* ≈ 100) of persistent CD4^+^ T-cell lymphopenia in the absence of human immunodeficiency virus 1 (HIV 1) infection have been reported so far. Of note, none of these patients have been associated with a specific defect of CD4 expression. Most of the cases display clinical manifestations that are characteristic of combined immunodeficiencies (15, 16). Although in the majority of the cases, the genetic etiology of Idiopathic T-CD4 lymphocytopenia (ICL) has not been investigated, preliminary molecular genetic studies in 20 patients suggest that, at least in some patients, there are mutations in several genes other than CD4 (i.e., RAG1, DOCK8, MAGT1), with pleotropic effects not restricted to CD4^+^ T-cells (17–19). Altogether, these findings suggest that the clinical and immunological alterations reported in ICL are most likely associated with a helper T-cell defect potentially combined with defects on other cell lineages, rather than with a lack of expression of the CD4 molecule.

Here we report for the first time in human a selective CD4 molecule deficiency associated with a homozygous autosomal recessive mutation in the CD4 gene that completely abrogates expression of the CD4 protein. The immunological and clinical features of this case support previous studies on CD4 KO mice suggesting that, although the immune response is affected in these cases, surrogate CD4-negative CD8-negative helper T-cells and Tregs can be produced in the absence of CD4 signaling, which are capable of replacing most of the functional roles of CD4^+^ T-cells.

## Case Presentation

**Figure 1:**
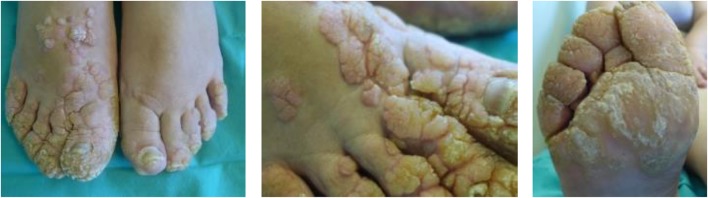
Photographs of warts present in the patient's feet.

A 45-year-old Caucasian female born to first-cousin parents, with two healthy children and without any relevant family history record of prior diseases, was seen at the service of Dermatology (University of Coimbra, Coimbra, Portugal) in March 2014 because of persistent extensive, skin-colored, exuberant, and disfiguring warts in both feet and hands since the age of 10 years (Figure 1). Warts were refractory to treatment with keratolytic agents, cryosurgery, and excision, with minor improvement after treatment with acitretin in association with topical 50% urea cream. Apart from this, the patient did not describe recurrent infection-related episodes or diseases, except for past medical history of measles and mumps during her infancy and varicella infection during her first pregnancy, which all resolved without complications. Of note, such past history of infections is not rare among the patient age-matched Portuguese population since vaccination for these diseases was introduced in the Portuguese national vaccination program years after she was born (1969): in 1974 for measles, in 1987 for mumps, and in 2004 for varicella (20–22). In fact, outbreaks of measles and mumps have been reported in Portugal until the late 80s to mid-90s, with peaks of >10,000 cases per year (20, 21).

In addition, she referred allergic rhino-conjunctivitis treated with cetirizine and fluticasone, and chronic polyarthralgias in the absence of impaired functionality. Serological studies were negative for (severe) infections, including HIV 1/2, HTLV-1, and syphilis. In turn, she showed IgG antibodies for ubiquitous pathogens including CMV >250 arbitrary units (AU/ml) (positive threshold >6AU/ml) and *Epstein–Barr* virus VCA = 192 U/ml (positive threshold >20 U/ml) and EBNA = 24 U/ml (positive threshold >20 U/ml) in the absence of serum IgM antibodies for these pathogens (0.06 AU/ml; positive threshold >6AU/ml). Slightly increased serum IgG levels (IgG: 1,430 mg/dl), associated with normal IgA (278 mg/dl), IgM (67 mg/dl), C3 (1.4 g/L), C4 (0.33 g/L), and C1 inhibitor (0.318 g/L) serum levels, were detected. In addition, anti-neutrophil and anti–double-strand DNA autoantibodies were negative, while antinuclear autoantibodies were weakly positive. Screening for immunological alterations by flow cytometry (Supplementary Material) (23–25) using the EuroFlow Primary Immunodeficiency Orientation Tube (PIDOT) (26, 27) showed an absolute defect of CD4-expressing T-cells (<0.01 cells/μl), with normal total T-cell, CD8^+^ TCRγδ^−^ T-cell, and NK-cell (absolute) numbers, associated with consistently increased B-cell counts vs. age-matched normal reference values. Importantly, (TCRαβ^+^ TCRγδ^−^) DN T-cells were significantly expanded (Table 1). Signs/symptoms associated with primary immunodeficiency other than persistent warts in the feet and hands were not observed either at presentation or during the subsequently 5-year follow-up period. Of note, peripheral blood (PB) monocytes and DCs showed no cell surface expression of CD4.

**Table 1:** Distribution of distinct populations of innate immune cells T- and B- lymphocytes in the CD4^null^ patient here reported compared to age-matched reference values.

**Leucocyte subsets**	**Patient**	**Age reference values**
T-cells	1,700 ± 733	(743–2,379)
CD4^+^ CD8^−^ TCRγδ^−^ T-cells	**0** **±** **0**	(501–1,654)
Naïve	**0** **±** **0**	(83–1,057)
Central memory/transitional memory	**0** **±** **0**	(235–784)
Effector memory	**0** **±** **0**	(25–208)
Terminally differentiated	**0** **±** **0**	(0–663)
CD4^−^ CD8^+^ TCRγδ^−^ T-cells	902 ± 367	(133–1,432)
Naïve	**556** **±** **226**	(29–386)
Central memory/transitional memory	217 ± 98	(59–453)
Effector memory CD27^−^	105 ± 26	(6–323)
Effector CD27^dim^	**4** **±** **6**	(7–457)
Terminally differentiated	33 ± 14	(0–500)
TCRγδ^+^ T-cells	**238** **±** **96**	(7–231)
CD4^−^ CD8^−^ TCRγδ^−^ T-cells	**560** **±** **287**	(4–24)
Treg-like (CD8^−^/TCRγδ^−^/CD25^++^/CD127^−^)	66	(22–141)^*^
TFH-like (CD8^−^/TCRγδ^−^/CXCR5^+^)	134	(45–240)^*^
Th1-like (CD8^−^/TCRγδ^−^/CXCR3^+^/CCR4^−^/CCR6^−^/CXCR5^−^)	139	(57–704)^*^
Th2-like (CD8^−^/TCRγδ^−^/CXCR3^−^/CCR4^+^/CCR6^−^/CXCR5^−^)	55	(24–123)^*^
Th17-like (CD8^−^/TCRγδ^−^/CXCR3^−^/CCR4^+^/CCR6^+^/CXCR5^−^)	43	(14–93)^*^
Th1/Th17-like (CD8^−^/TCRγδ^−^/CXCR3^+^/CCR4^−^/CCR6^+^/CXCR5^−^)	**146**	(20–124)^*^
NK-cells	603 ± 134	(150–672)
Classical monocytes	610 ± 258	(343–1,104)
CD62L^+^ cMo	249	(2–731)^*^
CD62L^−^ cMo	220	(19–473)
Non-classical monocytes (CD16^++^)	169 ± 102	(26–141)
iMo (CD14^+^/CD16^++^)	21	(0–89)
Late ncMo (CD14^−^/CD16^++^)	120	(0–160)
SLAN^−^ Late ncMo	96	(0–155)
SLAN^+^ Late ncMo	24	(0–52)
Plasmacytoid DCs	6 ± 3	(4–29)
Neutrophils	3,872 ± 787	(1,800–6,782)
Eosinophils	213 ± 64	(0–648)
Basophils	62 ± 15	(10–64)
B-cells	**517** **±** **241**	(48–413)
Immature B-cells	7.5 ± 2.1	(0.8–23)
Naïve B-cells	**394** **±** **155**	(26–244)
CD21^+^	**388** **±** **151**	(24–372)
CD21^−^	5.5 ± 3.5	(0.3–31)
Memory B-cells	**209** **±** **77**	(25–173)
CD27^+^	**194** **±** **71**	(19–160)
CD27^−^	15 ± 5.7	(1.4–17)
CD21^+^	**201** **±** **76**	(16–144)
CD21^−^	8.0 ± 1.4	(2.8–33)
IgM^++^D^+^	82 ± 38	(12–114)
IgG1^+^	**42** **±** **11**	(2.8–30)
IgG2^+^	**17** **±** **3.5**	(0.6–14)
IgG3^+^	**9.0** **±** **4.2**	(0.7–6)
IgG4^+^	0.8	(<0.01–4.1)
IgA1^+^	**44** **±** **18**	(2.5–27)
IgA2^+^	13 ± 1.4	(0.4–14)
Only IgD^+^	0.7 ± 1.0	(<0.01–0.9)
Plasmablasts	6.6 ± 6.2	(0.6–9.7)
IgM^+^	0.07	(0.04–1)
IgG1^+^	0.3	(<0.01–1.7)
IgG2^+^	<0.01	(<0.01–0.7)
IgG3^+^	0.08	(<0.01–0.2)
IgG4^+^	<0.01	(<0.01–0.1)
IgA1^+^	**6.2**	(0.2–3.8)
IgA2^+^	0.4	(0.04–2.9)
Only IgD^+^	<0.01	(<0.01–0.1)

*Results expressed as (mean ± standard deviation) absolute cell counts per μl of peripheral blood, obtained from repeated analysis performed during the last 5 years following diagnosis. Normal reference values expressed as minimum and maximum of age-matched healthy donors (40–59 years) based on previously published data (20, 21, 26, 28) are also shown between brackets (). Populations that showed abnormal absolute numbers compared to age-reference values are depicted in bold*.

* *Reference values obtained from CD4^+^ CD8^−^ TCRγδ^−^ T-cells of age-matched healthy donors. DNT, double-negative T-cell; Tregs, regulatory T-cells; TFH, follicular helper T-cells; DCs, dendritic cells; cMo, classical monocytes; iMo, intermediate monocytes; ncMo, non-classical monocytes*.

## Laboratory Investigations and Diagnostic Tests

### Expression of CD4 on T-Cells, Monocytes, and DCs

**Figure 2:**
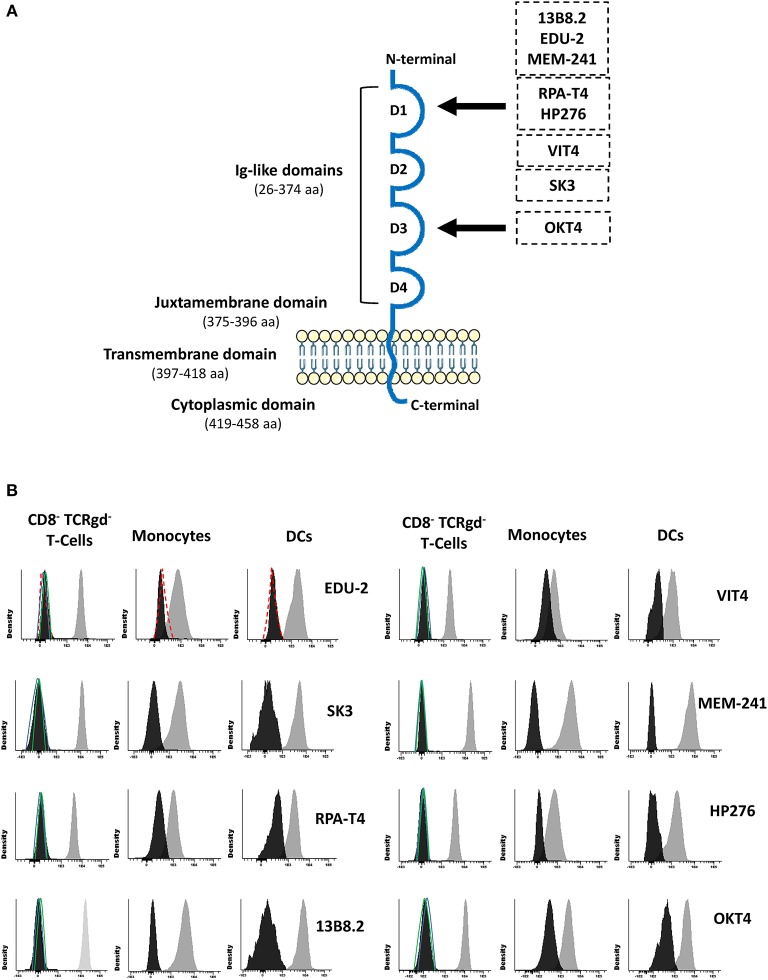
Schematic representation of the CD4 protein molecule **(A)** and CD4 surface membrane expression in the patient's (vs. healthy donor) blood CD8^−^ TCRγδ^−^ T-cells, monocytes, and dendritic cells **(B)**. Panel **(A)** shows a schematic representation of the CD4 protein molecule including the localization of epitopes identified by the distinct antibody clones used in this study (29–31). Amino acid positions that conform each domain are indicated between brackets. Black arrows depict the domain that contains those epitopes that the antibody clones are directed to. Panel **(B)** shows CD4 surface membrane expression levels for CD8^−^ TCRγδ^−^ T-cells, monocytes, and dendritic cells for the different anti-CD4 antibody clones tested in the patient (black histogram) compared to a representative healthy donor (gray histogram) and an isotype control (red dash line), and the staining for a negative population (CD8^+^ T-cells) in the patient (green line) and the healthy control (blue line). DCs, dendritic cells.

Since rare CD4 polymorphisms that abrogate reactivity of some monoclonal antibodies (MoAbs) with the CD4 molecule have been described (5, 6), CD4 expression was evaluated using eight different MoAb clones. These eight CD4 MoAb clones were directed against five different epitopes located in two distinct domains of the CD4 protein (Supplementary Material; Figure 2A), as confirmed in competitive staining inhibition experiments (data not shown), in line with previous data in the literature (32–34). Detectable levels of either surface membrane (sm) or intracytoplasmic (cy) CD4 expression were observed in none of the cell lineages that usually express this protein in blood of healthy controls, such as T-cells, monocytes, and DCs (Figure 2B). A lack of CD4 expression was confirmed for both classical T-cells and invariant MAIT and iNKT cells (data not shown). In contrast, the expression of other molecules previously associated with CD4 lymphopenia, such as CD3 and HLA-DR (35), was normal and comparable to that observed in healthy donors (data not shown). Besides no cellular CD4 protein being detected, normal soluble CD4 levels in plasma were observed in the patient using an ELISA assay with a pair of antibodies directed against the extracellular domains of CD4 (amino acids 26–390; Table 2).

**Table 2:** Soluble CD4 plasma levels detected in the patient and her children compared to two healthy donors.

	**CD4 concentration (ng/ml)**
Patient	7.61
Daughter	13.36
Son	10.64
Healthy donor 1	11.7
Healthy donor 2	9.20

### CD4 Gene DNA and cDNA Sequencing

**Figure 3:**
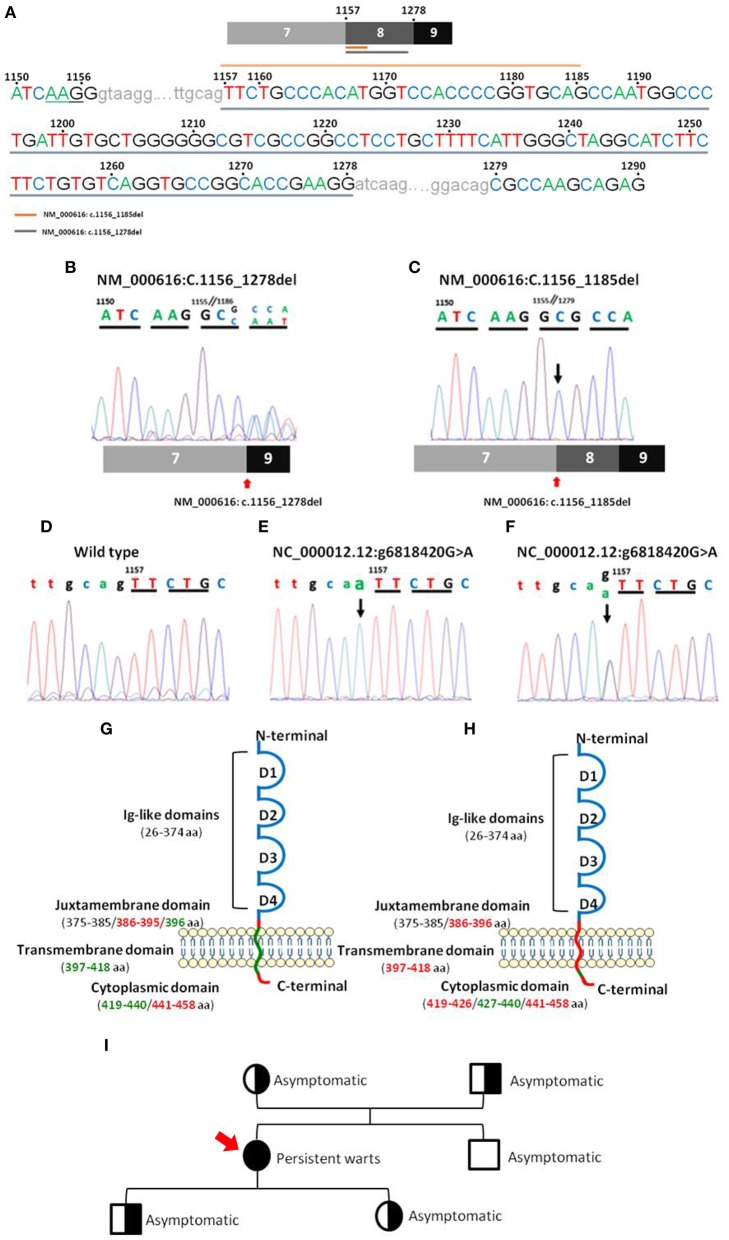
Nucleotide sequences of CD4 PCR products for the regions of interest and their localization in the CD4 gene. Panel **(A)** shows a representative sequence of CD4 focused on the region of interest. Gray underlining shows the nucleotide sequence affected by the major deletion (NM_000616: r.1157_1278del), and orange top line shows the nucleotide sequence affected by the minor deletion (NM_000616: r.1157_1185del). Panels **(B,C)** show mRNA electropherograms obtained from analysis of the minor (NM_000616: r.1157_1185del) and major (NM_000616: r.1157_1278del) deletions detected in the patient's CD4 cDNA, respectively, and the patient's relatives **(C)**. Panels **(D–F)** show genomic DNA electropherograms from: **(D)**, wild type CD4 gene; **(E)**, patient's homozygous mutated CD4 gene; and **(F)**, heterozygous mutated CD4 gene from the patient's parents and children. Panels **(G,H)** display a schematic representation of the localization in wild type CD4 protein of the mutated (green) nucleotide bases and the deleted (red) amino acid sequences, including the minor **(G)** and major **(H)** deletions, observed in the truncated CD4 cDNA molecules detected in the CD4^null^ patient here reported. Panel **(I)** shows the family pedigree (men are represented as squares and women as circles), including the key clinical manifestations and, between brackets, the results of CD4 DNA analysis in a coded/graphic format: half-shaded circles and squares, heterozygous DNA; fully shaded circles and squares, homozygous mutated DNA; and unshaded circles and squares, wild type DNA.

CD4 gene sequencing of patient DNA revealed an isolated homozygous mutation (Figure 3; Supplementary Table 1) in the last bp of the 7–8 intron (NC_000012.12: g6818420 G>A), corresponding to the juxtamembrane domain of the CD4 protein. This alteration was considered by the Variant Effect Predictor Tool (VEP) (36) as a splice acceptor variant with a high impact on CD4 protein transcription. No wild type CD4 DNA sequence was detected based on the analysis of the sequence of the amplicon products obtained after PCR amplification of DNA from the patient. Instead, two truncated forms of CD4 RNA/cDNA were detected. Both truncated forms of CD4 RNA presented with a frameshift deletion starting at the juxtamembrane region at the first bp of exon 8 and a premature stop codon associated with a truncated protein with normal extracellular domains in the absence of the anchoring domain to the membrane (Figure 3). The first frameshift deletion (NM_000616: c.1157_1278del) consisted of a complete deletion of exon 8 (122 pb), resulting in a 399-amino-acid protein. In turn, the second frameshift deletion produced a 430-amino-acid protein because of (only) a 29 bp deletion (NM_000616: c.1157_1185del) (Figure 3) that ended just before a 5 bp combination (TGCAG), homologous to the sequence observed at the end of the 7–8 intron (Figure 3).

In order to investigate the origin of the mutation, DNA from four first-degree patient relatives (all asymptomatic) was also analyzed. Thus, the mutation identified in the patient (NC_000012.12: g6818420 G>A) was also found in heterozygosis in DNA from each of her two children and each of her parents. In contrast, her brother's DNA only showed wild type CD4 gene sequences. Further cDNA sequencing from her children and her parents revealed two CD4 mRNA sequences, one carrying the large deletion observed in the patient (NM_000616: c.1156_1278del) and another with the unmutated (wild type) CD4 allele sequence (Figure 3). These results are fully consistent with a germinal mutation in the patient inherited from her parents and transmitted to her children. In contrast, in the patient's brother, both alleles were found to be wild type. Analysis of CD4 protein levels by flow cytometry confirmed that, although normal CD4^+^ T-cell counts were observed in the patient's children and parents (Table 3), the amount of expression of the CD4 protein on CD4^+^ T-cells, monocytes, and pDCs was reduced to around half when compared to healthy controls (*n* = 3) and the patient's brother, stained in parallel (Supplementary Table 2).

**Table 3:** PB distribution of distinct populations of innate immune cells T- and B-lymphocytes in the patient relatives as analyzed with the EuroFlow Primary Immunodeficiency Orientation Tube (PIDOT) vs. age-matched reference values.

	**Daughter^*mut*/*wt*^ (normal range)**	**Son^*mut*/*wt*^ (normal range)**	**Mother^*mut*/*wt*^ (normal range)**	**Father^*mut*/*wt*^ (normal range)**	**Brother^*wt*/*wt*^ (normal range)**
T-cells	1,470 (564–2,935)	1,159 (564–2,935)	1,561 (636–3,030)	1,910 (636–3,030)	1,056 (564–2,935)
CD4^+^	663 (207–1,900)	726 (207–1,900)	900 (345–1,474)	1,293 (345–1,474)	556 (207–1,900)
Naïve	288 (74–1,173)	129 (74–1,173)	145 (27–939)	251 (27–939)	**73** (74–1,173)
Central memory/transitional memory	307 (117–886)	421 (117–886)	634 (173–764)	630 (173–764)	370 (117–886)
Effector memory	53 (14–500)	174 (14–500)	118 (30–539)	410 (30–539)	90 (14–500)
Terminally differentiated	15 (0–87)	1.5 (0–87)	3.9 (0–219)	2.8 (0–219)	24 (0–87)
CD8^+^	611 (160–1,103)	370 (160–1,103)	567 (202–1,571)	487 (202–1,571)	335 (160–1,103)
Naïve	356 (33–737)	192 (33–737)	19 (2–223)	14 (2–223)	**30** (33–737)
Central memory/transitional memory	186 (54–424)	108 (54–424)	235 (42–321)	245 (42–321)	137 (54–424)
Effector memory	20 (2–515)	18 (2–515)	242 (11–925)	118 (11–925)	10 (2–515)
Effector CD27^dim^	1.1 (0–144)	1 (0–144)	27 (4–576)	11 (4–576)	5 (0–144)
Terminally differentiated	48 (1–273)	50 (1–273)	44 (0–905)	99 (0–905)	153 (1–273)
CD4^−^ CD8^−^ TCRγδ^−^	36 (5–79)	30 (5–79)	15 (2–27)	**73** (2–27)	8.7 (5–79)
TCRγδ^+^	160 (11–470)	33 (11–470)	80 (4–1,060)	57 (4–1,060)	156 (11–470)
NK-cells	233 (161–672)	242 (161–672)	195 (124–1,737)	733 (124–1,737)	**122** (161–672)
Neutrophils	5,468 (1,875–6,483)	**7,222** (1,875–6,483)	3,408 (1,904–5,516)	3,955 (1,904–5,516)	3,019 (1,875–6,483)
Eosinophils	133 (17–2,353)	82 (17–2,353)	468 (40–508)	**635** (40–508)	49 (17–2,353)
Basophils	36 (6–124)	43 (6–124)	42 (0–97)	**162** (0–97)	22 (6–124)
Monocytes	413 (198–1,048)	486 (198–1,048)	438 (201–840)	**1,480** (201–840)	363 (198–1,048)
Non-classical monocytes (CD16^++^)	91 (7–147)	109 (7–147)	188 (10–249)	**409** (10–249)	99 (7–147)
Plasmacytoid DCs	16 (2.7–27)	6.6 (2.7–27)	8.8 (4–19)	7.9 (4–19)	5.2 (2.7–27)

*Results expressed as absolute cell counts per μ of peripheral blood. Normal reference values are expressed as minimum and maximum values previously reported for age-matched healthy donors (20, 21). Altered cell numbers are depicted in bold. mut, mutated allele; wt, wild type allele, PB, peripheral blood*.

### Immunophenotypic and Functional Characterization of Expanded CD4/CD8 DN T-Cells

As described above, a lack of CD4 expression on patient T-cells was associated with abnormally expanded DN TCRγδ^−^ TCRαβ^+^ T-cell counts in PB (>30% of all T-cells; Table 1), compared to age-matched normal reference values (26, 27). Based on CD27, CD45RA, and CCR7 expression, these DN T-cells showed a similar distribution per maturation stage to that of CD4^+^ T-cells from age-matched healthy controls, both in relative and absolute numbers: (i) naïve: 27% and 240 cells/μ (6–66% and 83–676 cells/μl), (ii) central memory: 66% and 587 cells/μl (18–71% and 235–589 cells/μl), and (iii) effector memory: 7% and 62 cells/μl (2–66% and 14–221 cells/μl) (26, 27). In addition, expanded DN T-cells showed a polyclonal TCRVβ repertoire with a distribution per TCR family evaluated, fully consistent with reference TCRVβ repertoire values observed in HLA class II–restricted CD4^+^ T-cells from healthy donors (37) (Supplementary Figure 1). More extended phenotypic analysis of central/effector memory DN T-cells from the patient showed Treg and Th surrogate marker expression profiles for Tregs, TFH, Th1, Th2, Th17, and Th1/Th17 CD4^+^ helper T-cell to be present at frequencies similar to those observed for normal CD4^+^ T-cells (Table 1) from age-matched healthy donors (Botafogo et al., submitted).

Short-term *in vitro* stimulation, for 4 and 6 h, showed that the expanded DN T-cells were capable of producing cytokines from the main Th patterns (e.g., IFNγ, IL-4/IL-5, and IL-17A/IL-17F) at frequencies similar to CD4^+^ helper T-cells from age-matched healthy controls, when either polyclonal (i.e., PMA) or antigen-specific (i.e., CMV) stimuli that required antigen presentation were used (Table 4). In contrast, the percentage of DN T-cells expressing markers of cytotoxic T-cells (cyGranzyme B or cyPerforin) was decreased below <1% (normal age-matched range of <1–21% of DN T-cells).

**Table 4:** *In vitro* cytokine production by PB T-cells of the patient and her relatives after polyclonal (PMA + ionomycin) and after antigen-specific stimulation (whole CMV lysate).

**Cytokine**	**Subset**	**Healthy adults (*n* = 5)**	**Patient**	**Daughter**	**Son**	**Mother**	**Father**	**Brother**
**POLYCLONAL STIMULATION (PMA** **+** **IONOMYCIN)**								
IFNγ	CD8^+^ T-cells	26–67%	39%	26%	32%	81%	76%	76%
	CD4^+^ T-cells	19–32%	44%^*^	22%	45%	38%	27%	34%
IL-4/IL-5	CD8^+^ T-cells	0.1–0.3%	0.5%	0.1%	0.3%	0.4%	0.1%	0.6%
	CD4^+^ T-cells	1.6–2.9%	4%^*^	1.1%	2.6%	1.7%	1%	2.1%
IL-17A/IL-17F	CD8^+^ T-cells	0.2–1.3%	5%	0.7%	3.2%	6.3%	9%	5.7%
	CD4^+^ T-cells	1.4–2.5%	5%^*^	3%	14%	5.8%	6%	7.2%
**ANTIGEN-SPECIFIC STIMULATION (CMV)**								
IFNγ	CD8^+^ T-cells	0.3–0.7%	0.8%	0%	0.2%	1%	2.9%	0.3%
	CD4^+^ T-cells	0.5–5%	0.4%^*^	0.4%	4.8%	1.6%	1.6%	1%
IL-4/IL-5	CD8^+^ T-cells	0–0%	0%	0%	0%	0%	0%	0.6%
	CD4^+^ T-cells	0–0.2%	0.1%^*^	0%	0%	0%	0.2%	0%
IL-17A/IL-17F	CD8^+^ T-cells	0–0%	0%	0%	0%	0%	0%	0.05%
	CD4^+^ T-cells	0–0%	0.4%^*^	0%	0%	0%	0%	0.08%

*Results expressed as percentage of cells positive for each cytokine in CD8^+^, CD4^+^ T-cells and double-negative T-cells (DNTs)*.

* *Results from double CD4^−^ CD8^−^ TCRγδ^−^ T-cells*.

### Immunophenotypic and Functional Evaluation of Cytotoxic, Humoral, and Innate Immune Cells and Immune Responses

Normal total CD8+ T-cell, TCRγδ^+^ T-cell, and NK-cell counts were found in the patient blood, although the latter two were increased in some of the monitoring time points tested during the 5-year follow-up period. In contrast, consistently increased naïve CD8+ T-cell counts (Table 1) vs. age-matched reference values (26, 27), associated with normal central and effector memory CD8+ T-cell numbers, were found in the patient's blood at different time points. Expression of cytolytic enzymes (cyGranzyme B and cyPerforin) was detected in CD8^+^ T-cells (13 and 9% of CD8^+^ T-cells) and NK-cells (>99%) at normal values (15–65% and 10–53% of CD8^+^ T-cells, respectively; >99% of NK-cells). In addition, CD8^+^ TCRαβ^+^ T-cells also showed a normal polyclonal TCRVβ repertoire (vs. normal age-matched reference values, shown in Supplementary Figure 1) (37) and a normal cytokine production profile in response to PMA and CMV (Table 4) (29).

Detailed dissection of the PB B-cell compartment from the patient showed persistently increased total B-cell counts due to increased naïve (CD21^+^) B-cells, IgG_1−3_ and IgA_1_ memory B-cells, and IgA_1_ plasmablasts, with normal immature/transitional B-cell numbers (Table 1) (28, 38), in line with the observed higher serum IgG levels.

Analysis of circulating PB monocytes based on expression of CD14 (LPS receptor) and CD16 (low-affinity Fc IgG receptor) showed normal absolute counts for all subsets (Table 1) (39), including: (1) CD14^+^ CD16^−^ classical monocytes (cMo), (2) CD14^+^ CD16^+^ intermediate monocytes (iMo), and (3) CD14^−^ CD16^+^ non-classical monocytes (ncMo). Further dissection of cMo and ncMo based on the expression of the CD62L and SLAN selectins, respectively, did not show significant differences vs. normal age-matched reference values (39). In addition, CD4-negative monocytes and DCs were capable of producing cytokines at frequencies similar to their CD4^+^ counterparts from age-matched healthy control blood, after stimulation with LPS and γ-IFN (Supplementary Table 3) (40).

### Clinical Records and Immunophenotypic and Functional Features of Immune Cells of Patient Relatives

All patient relatives were completely asymptomatic, and they have no past history of recurrent infections or cancer. The father of the patient has had type diabetes since he was 45 years old. They do not have other records of autoimmunity. No significant consistent alterations were found as regards the distribution of immune cell subsets (*n* > 50) in the blood of the relatives of the patient, once compared to age-matched reference values, except for a slight increase in IgG_1_, IgG_4_, and IgD-only memory B-cells in the patient's daughter (Table 3 and Supplementary Tables 4, 5). In addition, a normal TCRvβ repertoire distribution (data not shown) together with normal *in vitro* cytokine production profiles in response to both PMA and CMV (Table 4) were observed among the patient's parents, children, and brother.

## Discussion

Here we describe for the first time in the literature a patient carrying an inherited homozygous autosomal recessive mutation in the CD4 gene leading to complete absence of CD4 expression on the surface membrane of blood T-cells, monocytes, and DCs. This was associated with a relatively mild clinical phenotype consisting of extensive (treatment-refractory) warts in both feet and hands. Lack of CD4 expression was confirmed by flow cytometry on both the surface and cytoplasm of T-cells (<0.01 CD4^+^ cells/μl) and other CD4^+^ myeloid immune innate cells (<0.01 CD4^+^ monocytes and DCs/μl). Despite this, normal soluble CD4 protein levels were found in plasma by ELISA. Rare CD4 polymorphisms that abrogate reactivity of some MoAbs with the CD4 molecule have been described (5, 6). However, in our patient, we demonstrated a lack of reactivity for eight different MoAb clones directed against five distinct epitopes located in two different domains of the CD4 protein, which confirms that the lack of CD4 expression was not due to any previously described single-nucleotide polymorphism in the CD4 gene (5, 6).

In contrast, CD4 gene sequencing of patient DNA revealed that the lack of CD4 expression at the cell surface membrane was associated with a homozygous mutation of the CD4 gene in the first bp of the 7–8 intron. This mutation affected downstream coding sequences corresponding to regions located between the juxtamembrane and transmembrane domains of the *CD4* gene. As a result of the mutation, two frameshift deletions from NM_000616: c.1157 onward were found in the mRNA/cDNA sequences of both alleles of CD4. In line with these findings, VEP analysis (36) predicted that the genomic alteration detected (NC_000012.12: g6818420 G>A) would cause the observed cDNA deletions due to modifications in the corresponding splicing site. Identification of a 5 bp sequence at the end of the minor deletion (NM_000616: c.1157_1185del), homologous to the end sequence of wild type intron 7, suggests that in this patient, a new alternative splicing variant might have occurred 29 pb downstream of the mutation. This might reflect a “repair” attempt to produce a functional CD4 protein capable of anchoring CD4 to the cell surface membrane. However, further studies are necessary to confirm this hypothesis and explain the presence of the minor deletion variant identified here at the mRNA level.

Further analysis of the patient's parents and children confirmed that the presence of the mutation identified in the patient was an inherited germline mutation (NC_000012.12: g6818420 G>A), as it was also found in heterozygosis in the two parents and in her two children. Thus, in all four family members two cDNAs were detected: a wild type and a truncated (NM_000616: c.1157_1278del) CD4 cDNA. Most interestingly, the presence of the mutation in heterozygosis resulted in decreased levels of CD4 protein expression in all CD4^+^ cell types to around half of normal CD4 levels per cell. This further confirms the direct association between the (NC_000012.12: g6818420 G>A) CD4 gene mutation and CD4 expression levels on immune cells in the blood for a codominant gene expression profile. In contrast, this or other CD4 gene mutations were not found in the brother of the patient, who only showed wild type cDNA and normal levels of cellular CD4 expression in blood T-cells, monocytes, and DCs.

Despite no cases of CD4^null^ mutation having been previously reported in humans, the immunological consequences of a lack of CD4 expression have been extensively analyzed in CD4 KO mice (13, 14, 41). Although it is well-established that CD4 plays an important role in differentiation, maturation, and the functionality of MHC/HLA class II–restricted T-lymphocytes, previous studies in CD4 KO mice indicate that this molecule is not absolutely required for (i) positive selection of T-cells (41, 42) and (ii) the effector function of MHC/HLA class II–restricted helper T-cells (13, 14, 41, 43). Thus, CD4 KO mice are apparently healthy, fertile, and indistinguishable from wild type littermates on gross physical inspection (13). In fact, except for a lack of CD4^+^ T-cells, defects in the *CD4* gene did not show a major impact on leukocyte production and differentiation, with normal CD8^+^ T-cell, B-cell, and myeloid cell counts in CD4 KO mice (13, 14, 41). Here, we confirmed that, also in our patient, apart from a lack of CD4^+^ T-cells and CD4^+^ innate cells, the CD4 mutation was associated with overall normal lymphocyte and myeloid cell counts, including normal monocyte and DC counts and functionality.

Failure to control infections has been observed in mice (and humans) that lack CD4^+^ T-cells, after sustained CD4^+^ T-cell depletion, or due to MHC/HLA class II defects (35, 44). In contrast, CD4 KO mice are able to control infections at levels similar to wild type animals for virtually all pathogens investigated, including viral (13, 45–47), bacterial (43, 48, 49), and parasite (14) infections. The ability of CD4 KO mice to mount normal immune responses has been consistently associated with a subset of MHC/HLA class II–restricted T-cells that emerge from the thymus as DN TCRγδ^−^ TCRαβ^+^ T-cells that fully replace the functional role of conventional CD4^+^ T-helper cells (13, 14, 41, 43). These DN T-cells are expanded in PB of CD4 KO mice (10–20% of the T-cell pool in blood) (13) and other immune tissues, including the spleen (14), lymph nodes (13, 41), and mucosa (50). Interestingly, expanded DN T-cells in CD4 KO mice have been shown to efficiently help to mount potent cytotoxic and humoral immune responses, including those involving immunoglobulin class-switch recombination (13, 41). Similar to CD4 KO mice, our patient also showed an expansion of DN T-cells. Like their CD4 KO mouse counterpart, DN T-cells from the CD4^null^ patient here reported were capable of producing Th1, Th2, and Th17 cytokines at similar levels to those of normal conventional CD4^+^ T-cells, in response to both unspecific and specific stimuli requiring antigen presentation. In addition, they showed identical expression profiles for Th surrogate markers, including markers of Tregs, TFH, Th1, Th2, Th17, and Th1/Th17, compared to age-matched normal CD4^+^ blood T-cells (26, 27) (Botafogo et al., submitted). Despite this, the patient persistently showed abnormally increased naïve CD8 T-cell numbers in the blood with an inverted helper/cytotoxic T-cell ratio (0.6 for total T-cells, 0.3 for naïve T-cells). Previous studies in mice have shown that positive selection of CD4^+^ T-helper thymocytes is around fivefold less efficient in the absence of CD4, due to inefficient positive selection (42). These results suggest that the lack of CD4 might provide a competitive advantage to the CD8 lineage in the thymus, consistent with the increased naïve CD8 T-cell numbers found in our patient. However, the clonal diversity of DN helper T-cells was not affected, and, like in CD4 KO mice (14), a polyclonal TCRVβ repertoire was found among DN T-cells, similar to that of conventional CD4^+^ helper T-cells. Such a polyclonal DN T-cell repertoire as found in our CD4-deficient patient (and in CD4 KO mice) is different from the typically restricted TCRVβ repertoire observed in DN T-cells from healthy mice and humans (51) and from autoimmune lymphoproliferative syndrome (ALPS) patients (52), which usually contain expanded clones of MHC/HLA class I–related T-cells. Of note, the lack of CD4 expression by DN T-cells did not appear to significantly affect differentiation of cytotoxic T-cells, since normal antigen-experienced memory and effector CD8^+^ T-cell and NK-cell counts associated with normal expression profiles of cytolytic enzymes were observed in PB except for borderline low TCRαβ^+^ CD8^+^ CD27^lo^ effector T-cells. Likewise, we did not observe any significant global effect of the CD4 defect on the humoral immune response, as normal (or even slightly increased) IgM, IgG_1−3_, and IgA_1−2_ memory B-cell and plasmablast subset counts (associated with normal or slightly increased serum antibody levels) were observed in our patient. These findings suggest that DN T-cells from our patient are also capable of supporting T-cell–dependent B-cell activation and immunoglobulin isotype switching, as previously reported for CD4 KO mice (41). Altogether, these results support the notion that CD4 is dispensable for commitment of thymocyte precursors to the helper T-cell lineage as long as the affinity threshold for positive selection is sustained (53). At the same time, they strongly argue against an essential role for CD4 in delivering a unique instructional signal for T-helper cell lineage commitment. Although no cell surface membrane or intracytoplasmic expression of CD4 protein was detected in our patient, we found expression of truncated CD4 mRNA and normal soluble CD4 plasma levels evaluated with MoAb against extracellular domains of the protein. Further studies would be necessary to characterize the amino acid sequence of this soluble CD4 protein. Altogether, these findings support the notion that (TCRγδ^−^ TCRαβ^+^) DN T-cells committed to the CD4 T-cell lineage/compartment are produced even in the absence of CD4 expression. These cells may overall contribute to maintaining normal immune surveillance based on otherwise normal primary and secondary immune responses associated with fast clearance of infection, against both intracellular and extracellular pathogens, as previously observed in KO mice (13, 14, 43, 45, 49). However, a few studies have previously reported a decreased ability of these DN T-cells to induce long-term cytotoxic memory cells and prevent viral persistence in specific situations (44, 54, 55), which might contribute to explaining the recurrent skin lesions observed in our patient. Further studies are necessary to elucidate the precise mechanisms involved in the overall higher naïve and memory B-cell counts and the presence of potentially dysfunctional helper and regulatory antigen-associated/specific DN T-cells.

While the function of CD4 on T-cells has been characterized in detail, the functional role of CD4 on human monocytes is much less understood (56). At present, it is well-established that monocytes do not express lck (an src-family kinase) that interacts with the intracellular domain of CD4 in T-cells. Thus, it has been suggested that CD4 activation via interaction with MCH-II might contribute to cytokine production and differentiation of human blood cMo into ncMo and macrophages (56, 57). In our patient, the lack of CD4 expression did not show a significant impact either on the PB counts of DCs, cMo, and ncMo or on the profile and amount of inflammatory cytokines produced by these cells after *in vitro* stimulation vs. age-matched controls (26, 39). However, further functional studies are still required to determine the impact of this mutation on the distinct subsets of monocytes' functionality, including their susceptibility to be infected by HIV.

In fact, the most striking sign of immunodeficiency found in our patient is the presence of extensive verrucous lesions since she was 10 years-old, which were resistant to treatment with topical keratolytic agents, cryosurgery, and excision. Despite no CD4 gene defect having been previously reported in humans, several case reports have previously described the association of persistent idiopathic CD4^+^ T-cell lymphopenia, in the absence of infection with HIV 1 or HTLV-1/2, and/or of a well-defined (primary or secondary) immunodeficiency disease, with recalcitrant warts and generalized verrucosis (58–64). Thus, more than half of idiopathic CD4^+^ T-cell lymphopenia cases present with mucocutaneous lesions (15, 16). Disseminated warts have also been frequently found in several autosomal recessive genetic defects that present with CD4 lymphopenia (e.g., RAG1, RHOH, MST1, CORO1A, DOCK8) (17–19). However, none of these genetic defects are specific for the CD4 T-cell lineage, and Idiopathic CD4 lymphopenia patients typically display additional clinical and immunological features in common with other combined immunodeficiencies (65–68). In fact, detailed analysis of idiopathic CD4 lymphopenia cases reported in the literature shows that many of them are not selective CD4 T-cell deficiencies, since they commonly show additional immunologic defects including decreased CD8^+^ T-cell, B-cell, and NK-cell counts in PB and/or low immunoglobulin levels (15, 16). In these patients, the presence of such immune alterations other than CD4 lymphopenia is associated with a higher risk of severe opportunistic infections and a greater mortality (15, 16). In contrast, patients with selective depletion of CD4^+^ T-cells but normal CD8^+^ T-cell and B-cell counts, as well as normal serum antibody levels, have been reported to display clinical manifestations restricted to cutaneous or genital infections (69, 70), in the absence of a broader susceptibility to more severe infections. Further studies are therefore required to understand the apparently close association between the lack of expression of CD4 and persistent and recurrent warts, which points out a still-unraveled (critical) role of the CD4 protein.

In summary, here we report for the first time ever a patient carrying a CD4 gene mutation that translates into complete abrogation of CD4 expression in multiple lymphoid and myeloid cell lineages, associated with recurrent, treatment-refractory warts in both hands and feet, in the absence of other clinically relevant manifestations of disease. In contrast to individuals depleted in CD4^+^ T-cells due to an external agent (e.g., HIV infection) or an associated combined immunodeficiency involving other immune cell compartments, defective CD4 expression was associated in our patient with milder immunological and clinical manifestations, except for refractory and recurrent skin lesions. This unique case provides insight about the role of CD4 in human T-cell differentiation, T-cell selection in the thymus, and effector T-helper and Treg functions that supports previous observations in CD4 KO mice. Altogether, our findings suggest that although CD4 contributes to MHC/HLA class II binding and signal transduction, it is not essential to generate MHC/HLA-II–restricted helper T-cells and T-helper–dependent cytotoxic and humoral immune responses.

## Data Availability Statement

The raw data supporting the conclusions of this manuscript will be made available by the authors, without undue reservation, to any qualified researcher.

## Ethics Statement

The studies involving human participants were reviewed and approved by Comité de Ética de la Investigación con medicamentos. del area de Salud de Salamanca. Written informed consent to participate in this study was provided by the participants' legal guardian/next of kin.

## Author Contributions

RF, MP-A, AO, and EF contributed to the conception and design of the study. RF, ICo, and EF collected and analyzed the clinical and serological information of the patient and relatives. MP-A, EB, ICr, JA, VB, and AP performed the flow cytometry data acquisition and data analysis. MJ-A and EB performed the molecular biology data acquisition and data analysis. RF, MP-A, JA, AP, JD, AO, and EF critically reviewed the data. RF, MP-A, EB, MJ-A, AO, and EF wrote the manuscript. All authors contributed to manuscript revision and read and approved the submitted version.

### Conflict of Interest

The authors declare that the research was conducted in the absence of any commercial or financial relationships that could be construed as a potential conflict of interest.
