# Supplementary material for: Complete Multilineage CD4 Expression Defect Associated With Warts Due to an Inherited Homozygous CD4 Gene Mutation
Source: Front Immunol. 2019 Nov 8;10:2502. doi: 10.3389/fimmu.2019.02502 (PMC6856949; doi:10.3389/fimmu.2019.02502)
Supplement: Supplementary file 1 [file Data_Sheet_1.pdf]

SUPPLEMENTARY MATERIAL AND METHODS

Ethics statement. This study was carried out according to the Declaration of Helsinki and written informed consent was obtained from the patient, their relatives and/or their legal representatives, as well as from healthy donors investigated, before being enrolled in the study, for biological sample collection, laboratory investigations and diagnostic tests and data publication.

Flow cytometry immunophenotypic studies. CD4 surface membrane (sm) and intracellular cytoplasmic (cy) expression was evaluated in EDTA-anticoagulant peripheral blood (PB) from the patient, their relatives and controls using 8 different clones of fluorochrome-conjugated CD4 monoclonal antibodies (MoAb) (Figure 2A): SK3 (PerCP-Cy5.5; Becton/Dickinson Biosciences –BD-, San Jose, CA), RPA-T4 (Horizon Violet 500; BD), 13B8.2 (Phycoerythrin -PE- Cy5; Cytognos SL, Salamanca, Spain), EDU-2 (Fluorescein Isothiocyanate –FITC-; Cytognos SL), VIT4 (VioGreen; Miltenyi Biotec, Bergisch Gladbach, Germany), MEM-241 (Allophycocyanin –APC-; Exbio, Vestec, Czech Republic), HP2/6 (PerCP-Cy5.5; Immunostep SL, Salamanca, Spain) and OKT4 (Brilliant Violet -BV- 510, Biolegend). FITC conjugated mouse IgG2a (Cytognos SL) was used as isotype control for EDU.2 CD4 MoAb. Anti-CD4 clones were combined with other markers for appropriate simultaneous identification of T-cells -CD3 APC or CD3 PerCP-Cy5.5, plus CD8 FITC and TCR γδ PECy7; BD), monocytes and DCs (CD14 APC-H7, BD; CD123 PE, Miltenyi Biotec; and HLA-DR Pacific Blue -PacB-, Biolegend). The distribution of major leukocyte subsets in blood in the patient and her relatives was evaluated using a large set of markers that included the following validated EuroFlow antibody panels: 1) Primary Immunodeficiency Orientation Tube (PIDOT) (23,24), 2) B-cell and plasmablast/plasma cell immunoglobulin heavy chain (IgH) isotype tube (IgM, IgD, IgG_{1-3}, IgA_{1-2}) (25,26), 3) T-chronic lymphoproliferative disorder (T-CLPD) (27) and 4) T CD4 cell immune monitoring tube (28). In addition, the TCRvβ repertoire of PB T-cells found in the patient and her relatives was analyzed using the IOTest Beta Mark TCR Repertoire Kit

(Beckman Coulter, Brea, CA), in combination with CD3 APC, TCRγδ PECy7, CD8 APC-H7 (BD) and CD4 PacB (Biolegend).

EuroFlow standard operating procedures (SOP) for sample preparation were used, as described in detail in www.euroflow.org. Evaluation of smCD4 expression, Th-surrogate markers, and the TCRvβ repertoire, was performed using the Euroflow SOP for surface membrane staining only (29). Briefly, 100 µL of whole PB was incubated with saturating amounts of the corresponding MoAb combination for 30 min in the dark (room temperature –RT-). Thereafter, 2 mL of FACS lysing solution (BD) diluted 1:10 (vol:vol) in distilled water was added to the cell suspension and the cells incubated for another 10 min at RT. Afterward, stained nucleated cells were centrifuged and washed in phosphate buffered saline containing 0.5% bovine serum albumin (PBS-BSA). Analysis of cyCD4, cyGranzyme B and cyPerforin, was performed following the EuroFlow SOP for simultaneous staining of surface membrane and cytoplasmic markers (29). Briefly, 100 µL of whole PB were incubated at RT with MoAbs against surface membrane markers, washed with PBS-BSA and incubated with solution A of the Fix&Perm reagent kit (An der Grub, Vienna, Austria). Then, cells were washed and incubated for 15 min at RT with solution B of Fix & Perm and the corresponding intracellular markers. After this incubation, stained cells were centrifuged and washed twice in PBS-BSA. For analysis of PIDOT and IgH-isotype tubes, the EuroFlow bulk-lysis SOP was used, as described in detail elsewhere (26,30) and in the EuroFlow website (www.EuroFlow.org). Briefly, up to 2mL of blood diluted in a total volume of 50 mL of an ammonium chloride hypotonic solution (1:25mL vol:vol per 50 ml tube), was gently mixed and incubated for 15 min in a roller. Then, nucleated cells were centrifuged and washed twice in PBS-BSA. Subsequently, cell surface membrane markers were stained on nucleated cells with the corresponding antibody mixtures, as described above in more detail.

Instrument set-up and calibration, and data acquisition were performed according to the EuroFlow SOP (29) (www.EuroFlow.org). All samples were measured in FACSCanto-II and LSR Fortessa X-20 flow cytometers (BD) using the FACSDiVa software (BD). For data analysis, the Infinicyt software (Cytognos SL) was used.

Cytokine production by T-cells after *in vitro* stimulation. Cytokine secretion by T-cells was investigated at the cytoplasmic level by flow cytometry, both after polyclonal and antigen-specific stimulation, as previously described (31-33). Briefly, heparinized PB samples were diluted 1:1 (vol:vol) with RPMI-1640 culture medium (BioWhittaker, Walkersville, MD) supplemented with 2 mM of L-glutamine (Gibco BRL, Gaithersburg, MD), to which 10 µg/mL of brefeldin A (Sigma, St. Louis, MO) was added to block cytokine secretion by PB leukocytes. For polyclonal stimulation conditions, samples were incubated with 25 ng/mL of phorbol 12-myristate 13-acetate (PMA; Sigma) plus 1 µg/mL of ionomycin (Sigma) for 4 h at 37 °C in a 5% carbon dioxide, 95% humidity, sterile atmosphere. To evaluate antigen-specific T-cell responses, samples were incubated with 5ug/mL of a whole cytomegalovirus (CMV) lysate (Advance Biotechnologies Inc, Columbia, MD), together with purified costimulatory MoAb directed against CD28 and CD49d (BD) at a final concentration of 1 µg/mL (6 h at 37 °C) in a 5% carbon dioxide, 95% humidity, sterile environment. An unstimulated sample aliquot (containing brefeldin-A in the absence of any stimuli) was processed in parallel in an identical manner. To analyze cytokine secretion by T cells, simultaneous staining for cytoplasmic cytokines and surface membrane markers was performed as described above, following the EuroFlow SOPs (www.euroflow.com) (29). The source and specificity of the MoAb used to detect intracytoplasmic human cytokines were as follows: anti-IFNγ-BV421, anti-IL4-APC, anti-IL5-APC and anti-IL17F-PE (BD) and anti-IL17A-PE (Biolegend). Stained samples were measured in a FACSCanto-II flow cytometer using the FACSDiVa software. For data analysis, the Infinicyt software was used.

Cytokine production by monocytes and DCs after *in vitro* stimulation. For the analysis of cytokine production by PB monocytes DCs, 1 ml of each heparin-anticoagulated PB sample was cultured as previously described (71). Briefly, the sample was diluted as described in the previous section and 100 ng/ml of lipopolysaccharide (LPS, from Escherichia coli, serotype 055:B5; Sigma) and 10 ng/ml of human recombinant interferon (IFN)- (Promega, Madison, WI) were added as stimulants, in addition to RPMI supplemented with L-glutamine and brefeldin. An unstimulated sample aliquot, containing BFA in the absence of both LPS and IFN-, was processed in parallel, in an identical way. Afterward, PB samples were incubated for 6 hours at 37°C in a 5% CO2 and 95% humidity sterile environment. To analyze cytokine secretion by T cells, simultaneous staining for cytoplasmic cytokines and surface membrane markers was performed as described above, following the EuroFlow SOPs (www.euroflow.com) (26). The source and specificity of the MoAb used to detect intracytoplasmic human cytokines were as follows: anti-TGFβ-BV421, anti-IL12-BV421, anti-IL1β-PE, anti-IL13-PerCPC5.5, anti-TNFα-PerCPC5.5, anti-IL8-PECy7 and anti-IL10 PECy7 (BD) and anti-IL6-PE (Biolegend). Stained samples were measured in a FACSCanto-II flow cytometer using the FACSDiVa software. For data analysis, the Infinicyt software was used.

Determination of soluble CD4 plasma levels. Plasma samples from the patient, her relatives and two healthy donors were obtained by sequential centrifugation of EDTA-PB (800g for 10 min) and platelet-rich plasma (2000g for 5min). Once obtained, plasma was immediately stored at -80ºC until analyzed. Soluble CD4 plasma levels were measured using a commercially available Enzime-Linked Immunoabsorbent Assay (ELISA) assay and kit (SEB167Hu, Cloud-clone Co, Houston, TX).

Analysis of CD4 mRNA and DNA sequences. RNA extraction from blood of the patient and her relatives was performed at the Spanish National DNA Bank Carlos III, University of Salamanca, Salamanca, Spain; (http://www.bancoadn.org), following established standard protocols. Subsequently, cDNA was synthesized from 1µg of total RNA using the High Capacity cDNA

Reverse Transcription Kit (Applied Biosystems™, Foster City, CA). For mRNA sequencing, cDNA was sequentially amplified by conventional PCR (35 amplification cycles) and sequenced with a combination of pairs of primers (Supplementary Table 1) that cover all the coding CD4 mRNA sequence (NM_000616: ENST00000011653.8).

In parallel, DNA was also extracted from the patient and her relatives using the GeneJet Whole Blood Genomic DNA Purification Mini Kit (Thermo Fisher Scientific Baltics UAB, Vilnius, LT), following the recommendations of the manufacturer. To analyze exon 7, 8 and 9 sequences, as well as their intronic flanking regions –for a total of 2.130 base pairs (bp)-, the same strategy as described above was used with specific pairs of primers designed for the whole DNA sequence (Supplementary Table 1). All PCR products were checked by conventional electrophoretic procedures in a 1.5% agarose gel, sequenced in an automatic ABI Prism 3130*xl* Genetic Analyzer (Applied Biosystems), and the sequences obtained were analyzed with the Chromas 2.6.6 software (Technelysium Pty Ltd, South Brisbane QLD, AU).
