# Supplementary material for: Complete Multilineage CD4 Expression Defect Associated With Warts Due to an Inherited Homozygous CD4 Gene Mutation
Source: Front Immunol. 2019 Nov 8;10:2502. doi: 10.3389/fimmu.2019.02502 (PMC6856949; doi:10.3389/fimmu.2019.02502)
Supplement: Supplementary file 3 [file Table_1.pdf]

Supplementary Table 1. Sequences of the mRNA and DNA pairs of primers used in this study and their corresponding amplicon length and annealing temperature.

mRNA primers			
Name	Sequence 5'	Amplicon length (bp)	Annealing Temperature
cDNA_CD4-1Fw	ATGAACCGGGGAGTCCCTTT	479	55ºC
cDNA_CD4-1Rv	GCAATGTAGGAGTCCAAGGGG		
cDNA_CD4-2Fw	CCTGGTAGTAGCCCCTCAGT	379	55ºC
cDNA_CD4-2Rv	TACCCAGGACCCTAAGCTCC		
cDNA_CD4-3Fw	AAAACGGGTTACCCAGGACC	268	55ºC
cDNA_CD4-3Rv	ACAAGGAGGCAAAGGTCTCG		
cDNA_CD4-4Fw	GAGCCACTCAGCTCCAGAAA	420	55ºC
cDNA_CD4-4Rv	TCAGAAGACATGTAGCCCCA		

DNA primers			
Name	Sequence 5'	Amplicon length (bp)	Annealing Temperature
DNA_CD4-1Fw	ACCCCAAACGCTGTTTTTC	461	55ºC
DNA_CD4-1Rv	ACACCATACCCATAACCCC		
DNA_CD4-2Fw	AGAAGTGATGAAGTGAGGTGG	302	58ºC
DNA_CD4-2Rv	TGAGAGTGTGGGTGTGAGTG		
DNA_CD4-3Fw	CACACACATCACTCACACAC	522	55ºC
DNA_CD4-3Rv	GCATATCCATATCCAGGGCAC		
DNA_CD4-4Fw	CATGCACTCACACACACAGC	500	55ºC
DNA_CD4-4Rv	GCAGCAAGGGAACTTACGAG		
DNA_CD4-5Fw	GCCATGTAACTGCTTCTCC	382	52ºC
DNA_CD4-5Rv	ATCTTTTCCCACCCCTCTATC		

Supplementary Table 2. Normalized CD4 expression levels in $CD4^+$ T-cells, monocytes and dendritic cells of the patient's relatives, using different anti-CD4 clones.

Anti-CD4 Clone	Subset evaluated	Daughter (mut/wt)	Son (mut/wt)	Mother (mut/wt)	Father (mut/wt)	Brother (wt/wt)
**SK3**	**$CD4^+$ T cells**	65%	79%	62%	63%	100%
	**Monocytes**	82%	75%	59%	63%	97%
	**DCs**	39%	68%	73%	34%	95%
**RPA-T4**	**$CD4^+$ T cells**	71%	82%	92%	75%	100%
	**Monocytes**	67%	69%	71%	84%	100%
	**DCs**	76%	71%	74%	46%	100%
**13B8.2**	**$CD4^+$ T cells**	73%	100%	60%	66%	NA
	**Monocytes**	62%	78%	58%	67%	NA
	**DCs**	77%	89%	63%	38%	NA
**EDU-2**	**$CD4^+$ T cells**	55%	65%	42%	51%	NA
	**Monocytes**	62%	61%	51%	67%	NA
	**DCs**	65%	62%	56%	32%	NA
**VIT4**	**$CD4^+$ T cells**	65%	21%	ND	ND	NA
	**Monocytes**	72%	80%	ND	ND	NA
	**DCs**	73%	65%	ND	ND	NA
**MEM-241**	**$CD4^+$ T cells**	67%	80%	57%	66%	100%
	**Monocytes**	55%	56%	54%	63%	100%
	**DCs**	73%	74%	67%	35%	89%
**HP276**	**$CD4^+$ T cells**	65%	76%	55%	62%	NA
	**Monocytes**	58%	59%	55%	66%	NA
	**DCs**	71%	66%	62%	34%	NA
**OKT4**	**$CD4^+$ T cells**	73%	54%	60%	63%	100%
	**Monocytes**	57%	56%	62%	73%	100%
	**DCs**	66%	71%	69%	39%	100%

Results expressed as percentage of median fluorescence intensity (MFI) normalized by the median CD4 MFI levels obtained in 3 healthy donors –(MFI of $CD4^+$ T-cells/MFI of normal HD $CD4^+$ T-cells) x100-. CD4 expression was undetectable in all peripheral blood leucocyte subsets from the $CD4^{null}$ patient. NA: Not available; DCs: dendritic cells. mut: mutated allele. wt: wildtype allele.

Supplementary Table 3. ***In vitro*** **cytokine production by PB monocytes and dendritic cells of the patient after (LPS+γIFN) stimulation compared to an age-matched healthy adult processed in parallel.**

Cytokine	Subset	Healthy adult	Patient
**IL-1β**	**Monocytes**	89%	90%
	**DCs**	<1%	<1%
**IL-6**	**Monocytes**	90%	90%
	**DCs**	3%	6%
**IL-8**	**Monocytes**	84%	93%
	**DCs**	3%	9%
**IL-10**	**Monocytes**	3%	1%
	**DCs**	<1%	<1%
**IL-12**	**Monocytes**	14%	22%
	**DCs**	2%	8%
**IL-13**	**Monocytes**	<1%	<1%
	**DCs**	<1%	<1%
**TNFα**	**Monocytes**	90%	90%
	**DCs**	5%	10%
**TGFβ**	**Monocytes**	8%	7%
	**DCs**	<1%	<1%

Results expressed as percentage of cells positive for each cytokine within monocytes and dendritic cells (DCs).

Supplementary Table 4. Absolute number of central/effector memory DN T-cells in peripheral blood based on the expression of Th surrogate markers in the patient´s relatives compared to age-matched healthy donors.

	**Daughter$^{mut/wt}$ (normal range)**	**Son$^{mut/wt}$ (normal range)**	**Mother$^{mut/wt}$ (normal range)**	**Father$^{mut/wt}$ (normal range)**	**Brother$^{wt/wt}$ (normal range)**
**CD4-CD8-**					
**Tregs**	74 (30-95)	55 (30-95)	109 (31-132)	122 (31-132)	52 (30-95)
**TFH**	56 (55-150)	61 (55-150)	176 (68-325)	156 (68-325)	112 (55-150)
**Th1**	92 (27-116)	**156** (27-116)	98 (45-587)	91 (45-587)	86 (27-116)
**Th2**	32 (16-70)	36 (16-70)	79 (25-220)	147 (25-220)	55 (16-70)
**Th17**	40 (14-45)	35 (14-45)	50 (21-120)	99 (21-120)	43 (14-45)
**Th1/17**	44 (23-115)	**153** (23-115)	141 (12-236)	98 (12-236)	63 (23-115)

Results expressed as absolute cell counts per µL of peripheral blood. Normal reference values are expressed as minimum and maximum for age-matched healthy donors (25). Cell populations with altered absolute cell counts are depicted in bold. mut: mutated allele. wt: wildtype allele.

Supplementary Table 5. Absolute counts for distinct PB B-cell subpopulations, including memory B-cells and plasmablasts expressing different IgH isotypes and isotype subclasses in the patient's relatives compared to age-matched healthy donors.

	Daughter$^{mut/wt}$ (normal range)	Son$^{mut/wt}$ (normal range)	Mother$^{mut/wt}$ (normal range)	Father$^{mut/wt}$ (normal range)	Brother$^{wt/wt}$ (normal range)
**B-cells**	467 (41-470)	375 (41-470)	198 (42-384)	83 (42-384)	131 (41-470)
**Immature B-cells**	2 (0.25-24)	**29** (0.25-24)	**0.2** (0.69-36)	3 (0.69-36)	2.3 (0.25-24)
**Naïve B-cells**	191 (13-288)	204 (13-288)	108 (15-280)	55 (15-280)	71 (13-288)
**CD21$^+$**	183 (13-284)	203 (13-284)	103 (14-280)	52 (14-280)	203 (13-284)
**CD21$^-$**	8.4 (0.2-9.1)	1.9 (0.2-9.1)	4.5 (0.5-32)	3 (0.5-32)	3.8 (0.2-9.1)
**Memory B-cells**	**267** (23-221)	140 (23-221)	88 (8.1-128)	24 (8.1-128)	52 (23-221)
**CD27$^+$**	**246** (20-204)	130 (20-204)	76 (12-116)	21 (12-116)	48 (20-204)
**CD27$^-$**	18 (1.2-21)	7.3 (1.2-21)	11 (0.6-16)	1.7 (0.6-16)	3.7 (1.2-21)
**CD21$^+$**	237 (19-201)	130 (19-201)	79 (12-111)	19 (12-111)	46 (19-201)
**CD21$^-$**	27 (1.9-28)	7.3 (1.9-28)	9 (0.8-33)	3.2 (0.8-33)	5.3 (1.9-28)
**IgM^{++}D$^+$**	159 (7.9-122)	76 (7.9-122)	20 (2-72)	10 (2-72)	25 (7.9-122)
**IgG1$^+$**	**55** (3.2-40)	29 (3.2-40)	23 (1.3-31)	3.2 (1.3-31)	12 (3.2-40)
**IgG2$^+$**	8 (1.6-30)	10 (1.6-30)	7 (0.4-12)	0.5 (0.4-12)	3.9 (1.6-30)
**IgG3$^+$**	7 (0.5-8.4)	3.7 (0.5-8.4)	5 (0.4-8.1)	1.3 (0.4-8.1)	2.0 (0.5-8.4)
**IgG4$^+$**	**2.7** (<0.01-2.4)	2.1 (<0.01-2.4)	1.1 (<0.01-2.1)	0.2 (<0.01-2.1)	0.7 (<0.01-2.4)
**IgA1$^+$**	21 (2.1-43)	11 (2.1-43)	21 (1.7-25)	4.8 (1.7-25)	6 (2.1-43)
**IgA2$^+$**	7 (1.2-18)	5 (1.2-18)	7.7 (0.6-11)	2 (0.6-11)	2.6 (1.2-18)
**Only-IgD$^+$**	**2.9** (<0.01-2.4)	0.8 (<0.01-2.4)	**2** (<0.01-0.2)	0.2 (<0.01-0.2)	<0.01 (<0.01-2.4)
**Plasmablasts**	6.4 (1.1-25)	1.9 (1.1-25)	1.4 (0.14-18)	0.9 (0.14-18)	1.3 (1.1-25)
**IgM$^+$**	0.9 (0.05-4.7)	0.1 (0.05-4.7)	0.05 (0.01-0.8)	0.04 (0.01-0.8)	0.03 (0.05-4.7)
**IgG1$^+$**	0.7 (0.05-4.4)	0.2 (0.05-4.4)	0.02 (0.01-0.6)	0.09 (0.01-0.6)	0.08 (0.05-4.4)
**IgG2$^+$**	0.2 (<0.01-2.6)	0.2 (<0.01-2.6)	<0.01 (<0.01-1.6)	<0.01 (<0.01-1.6)	0.1 (<0.01-2.6)
**IgG3$^+$**	<0.01 (<0.01-0.3)	<0.01 (<0.01-0.3)	<0.01 (<0.01-0.2)	<0.01 (<0.01-0.2)	<0.01 (<0.01-0.3)
**IgG4$^+$**	0.04 (<0.01-0.4)	0.02 (<0.01-0.4)	<0.01 (<0.01-0.1)	<0.01 (<0.01-0.1)	<0.01 (<0.01-0.4)
**IgA1$^+$**	1.7 (0.3-6.9)	0.9 (0.3-6.9)	0.7 (0.004-3.3)	0.5 (0.04-3.3)	0.4 (0.3-6.9)
**IgA2$^+$**	1.7 (0.2-4.2)	0.2 (0.2-4.2)	0.4 (0.06-1.2)	0.2 (0.06-1.2)	0.6 (0.2-4.2)
**Only-IgD$^+$**	0.1 (<0.01-1.1)	0.02 (<0.01-1.1)	<0.01 (<0.01-0.2)	<0.01 (<0.01-0.2)	<0.01 (<0.01-1.1)

Results expressed as absolute cell counts per µL of peripheral blood. Normal reference values are expressed as minimum and maximum for age-matched healthy donors (23). Cell populations with altered absolute cell counts are depicted in bold. mut: mutated allele. wt: wildtype allele.
